# Genome-wide identification of aquaporin family in *Camellia oleifera* and their response characteristics to drought stress

**DOI:** 10.3389/fpls.2026.1959769

**Published:** 2026-09-28

**Authors:** Mengqi Lu, Kui Liu, Qizouhong He, Shaofeng Peng, Yanling Zeng, Xiaofeng Tan, Yongzhong Chen, Longsheng Chen, Kai Wang

**Affiliations:** 1 State Key Laboratory of Woody Oil Resources Utilization, Hunan Academy of Forestry, Changsha, China; 2 Yuelushan Laboratory, Changsha, China; 3 National Engineering Research Center of Youcha, Hunan Academy of Forestry, Changsha, China; 4 State Key Laboratory of Woody Oil Resources Utilization, Central South University of Forestry and Technology, Changsha, China

**Keywords:** aquaporin, *Camellia oleifera*, drought stress, expression analysis, gene structure

## Abstract

Aquaporins (AQPs) facilitate transmembrane water transport and are essential for plant growth, development, and stress adaptation. *Camellia oleifera* is an important woody edible oil tree. Drought stress frequently occurs during the rapid fruit expansion and maturation stages, and AQPs might mediate its drought response. However, the diversity, molecular characteristics and biological functions of *AQPs* in *C. oleifera* remain largely uncharacterized. Here, 166 *CoAQPs* were identified and classified into five subfamilies based on phylogenetic analysis. They were mainly localized to the plasma membrane and vacuole. Members within the same subfamily shared conserved gene structures and motif compositions. Segmental duplication was the main driving force for *CoAQPs* expansion. The promoter region of *CoAQPs* contained abundant cis-acting elements associated with light response, phytohormone response, stress response, and plant growth and development. Tissue-specific expression analysis revealed that 50 *CoAQPs* were highly expressed in leaves, whereas 27 *CoAQPs* showed differential expression in developing seeds. Drought stress induced significant physiological and biochemical responses in leaves and fruits of *C. oleifera*. Transcriptome data and real-time quantitative PCR analyses indicated *CoAQPs* extensively participated in drought response. This study will lay a theoretical foundation for exploring water metabolism mechanisms and screening gene resources for stress tolerance and quality improvement in *C. oleifera*.

## 1 Introduction

Water is abundant in plant cells and plays vital roles in substance metabolism, energy transfer and stress adaptation. Aquaporins (AQPs) belong to the major intrinsic proteins (MIP) superfamily. AQPs are widely present in various plant tissues and can not only facilitate transmembrane transport of water molecules, but also effectively transport other small molecules such as glycerol, CO_2_, ammonia, urea, boric acid, and H_2_O_2_ (Maurel et al., 2009; Li et al., 2008; Uehlein et al., 2012; Di Giorgio et al., 2016; Guo et al., 2022; Maurel et al., 2015; Alam et al., 2026). To date, the AQP family has been systematically characterized in multiple plants, such as *Oryza sativa* containing 33 *OsAQPs*, *Arabidopsis thaliana* containing 35 *AtAQPs*, and *Populus trichocarpa* containing 55 *PtAQPs* (Maurel et al., 2015; Lopez et al., 2012). Based on structural and sequence characteristics, the AQPs are classified into five major subfamilies, including plasma membrane intrinsic proteins (PIPs), tonoplast intrinsic proteins (TIPs), nodulin26-like intrinsic proteins (NIPs), small basic intrinsic proteins (SIPs), and uncategorized X intrinsic proteins (XIPs) (Maurel et al., 2015; Lopez et al., 2012). Most AQP members function as efficient water channels with additional substrates such as glycerol, CO_2_ and H_2_O_2_ for PIPs, glycerol, urea, H_2_O_2_ and NH_3_ for TIPs, metalloids, H_2_O_2_, NH_3_ and lactic acid for NIPs, or metalloids and H_2_O_2_ for XIPs (Maurel et al., 2015; Beamer et al., 2021). However, XIPs are absent in monocots and Brassicaceae such as *O. sativa*, and *A. thaliana* (Lopez et al., 2012; Maurel et al., 2015), which may be attributed to multiple independent loss events during plant evolution.

AQPs are involved in plant growth and development, such as seed development and germination (Gattolin et al., 2011), root growth (Reinhardt et al., 2016), stomatal movement (Uehlein et al., 2008), fruit development (Wang et al., 2017), and pollen development (Di Giorgio et al., 2016). Tomato *MdPIP1;3* was an efficient water transport gene, and the growth rate of its transgenic tomato fruit was faster, and the fruit sizes and fresh weights were bigger (Wang et al., 2017). The pollen-specific *AtNIP4;1* and *AtNIP4;2* in *A. thaliana* can transport water, ammonia, boric acid, and thus regulated pollen germination and pollen tube growth (Di Giorgio et al., 2016). Furthermore, AQPs participate in responses to abiotic and biotic stresses, including drought, salt, cold, and diseases (Guo et al., 2022; Lu et al., 2018; Zhou et al., 2014; Tian et al., 2016). For example, overexpression of soybean *GmPIP2;9* increased intercellular CO_2_ concentration and stomatal conductance in leaves, thereby improving photosynthetic capacity and drought tolerance of plants (Lu et al., 2018). Under salt stress, the expression of soybean *GmPIP1;6* was upregulated, and its overexpression lines exhibited enhanced leaf gas exchange and carbon assimilation efficiency, as well as improved root hydraulic conductivity (Zhou et al., 2014). Some *AQPs* dually participate in the regulation of plant growth and stress tolerance. For example, *GmPIP1;6* improved soybean yield and salt tolerance (Zhou et al., 2014), whereas *MdPIP1;3* promoted fruit expansion and enhanced cold tolerance in apple (Wang et al., 2017). Generally, functional characterization of *AQPs* is indispensable. It helps unravel the molecular mechanisms of plant growth and stress responses, and provides valuable gene resources for stress-tolerance molecular breeding.

*Camellia oleifera* Abel. is a unique woody edible oil tree in China, with great economic and ecological values (Zhuang, 2008). Seasonal drought acts as a major abiotic stress that impairs plant growth, fruit development and oil accumulation in *C. oleifera*. Long-term drought can lead to a decrease in water content, wilting of leaves, and reduced photosynthetic efficiency. It also inhibits fruit development, and may eventually cause leaf abscission, fruit drop and even plant death (Zhang and Liao, 2026; Liu et al., 2026; He et al., 2020). Current studies on drought response of *C. oleifera* mainly focus on physiological characteristics and transcriptome analysis (He et al., 2020; Dong et al., 2020; He et al., 2005). For AQPs, only a few PIP members have been preliminarily characterized, which may be involved in regulating fruit size and seed desiccation during late maturation (Hu et al., 2008; Pan, 2024). However, the roles of AQPs in drought response of *C. oleifera* remain largely uncharacterized. In this study, we performed a genome-wide identification of *AQP* gene family in *C. oleifera*, and systematically characterized its gene structure, evolutionary characteristics, and cis-acting elements in promoter regions. Combined with transcriptome data and real-time quantitative PCR (RT-qPCR), we identified key candidate genes involved in water regulation in leaves and seeds under drought stress, providing a foundation for subsequent functional characterization.

## 2 Materials and methods

### 2.1 Identification and phylogeny of *CoAQPs*

The Hidden Markov Model (HMM) of the MIP domain (PF00230) was downloaded from the InterPro database (https://www.ebi.ac.uk/interpro/). Candidate *AQP* genes containing this domain were retrieved from the *C. oleifera* genome (Tan, 2023) using the Simple HMM Search program in TBtools (Chen et al., 2023), with an E-value cutoff of 1 × 10^-10^. For genes with multiple alternatively spliced transcripts, only the longest transcript was retained for analyses. The integrity of the MIP domain was further verified by the CD-Search tool (https://www.ncbi.nlm.nih.gov/Structure/cdd/wrpsb.cgi), and thus the members of *CoAQP* gene family in *C. oleifera* were ultimately confirmed. Based on sequence homology and phylogenetic relationships with AQP members from *A. thaliana* (Johanson et al., 2001) and *P. trichocarpa* (Lopez et al., 2012), the *CoAQP* genes were named. The maximum likelihood phylogenetic tree was constructed using MEGA11 under JTT+G5 model with 1000 bootstraps, and subsequently visualized and modification using the iTOL server (https://itol.embl.de/).

### 2.2 Characterization and structure of *CoAQPs*

The molecular weight, theoretical pI, instability index and grand average of hydropathicity was predicted by the ProtParam program (https://web.expasy.org/protparam/). The transmembrane (TM) domains and potential phosphorylation sites were predicted by the TMHMM-2.0 program (https://services.healthtech.dtu.dk/services/TMHMM-2.0/) and NetPhos-3.1 service (https://services.healthtech.dtu.dk/services/NetPhos-3.1/). The subcellular localization was predicted by the Plant-mPLoc (http://www.csbio.sjtu.edu.cn/bioinf/plant-multi/) and WoLF PSORT (https://wolfpsort.hgc.jp/). The conserved motifs were discovered by the MEME program (https://meme-suite.org/meme/tools/meme), with the number of motifs set to ten and an E-value threshold of 1e-10. The motifs and exon-intron structure were visualized with the TBtools software (Chen et al., 2023).

### 2.3 Chromosome localization, collinearity analysis, and cis-acting element analysis

The gene position on chromosomes was visualized with the TBtools software (Chen et al., 2023). The analysis of segmental duplication (SD) and tandem duplication (TD) events, and the ka/ks value calculation of gene pairs was performed by the TBtools software (Chen et al., 2023). The 2000 bp sequence upstream of the transcription start site (ATG) was extracted as the promoter, and the cis-acting element were analyzed by the PlantCare program (https://bioinformatics.psb.ugent.be/webtools/plantcare/html/).

### 2.4 RNA‐seq data collection and analysis

Tissue-specific expression of *CoAQPs* were analyzed using transcriptome data from pollen (Zhou et al., 2020), leaves (Xia et al., 2014), roots (Wang, 2024), and seeds at different developmental stages (Zhang et al., 2021). Expressions of *CoAQPs* in *C. oleifera* leaves under drought stress (Lu et al., 2025) were analyzed. The drought treatment and sample collection were performed as described below. The *C. oleifera* seedlings were subjected to natural drought stress, with normal irrigation treatment as the control group (CK). Leaves were harvested on days 2, 4, and 6 after drought treatment for transcriptome analysis. The expression patterns of *CoAQPs* were visualized by heatmaps. Given that the transcriptome datasets were derive from different studies, to minimize potential technical bias, only within-dataset relative expression comparisons were performed, and cross-dataset comparisons were limited to qualitative expression trends.

### 2.5 Drought treatment and indicator examination

According to the method described by Lu et al. (2025), nine four-year-old potted *C. oleifera* seedlings of bearing fruits were selected in August 2025 and randomly divided into three groups (three seedlings per group), representing three biological replicates. All potted seedlings were maintained under a rain shelter in an outdoor environment. At the onset of the experiment (Day 0 d), all pots were watered to field capacity, after which no further watering was carried out to initiate the natural drought experiment. Plant phenotypic observations and photography were performed on days 0, 2, 4, and 6 of drought treatment. The relative soil water content was determined by a soil water analyzer (FALA Electronic, Xuzhou, China) (**Supplementary Table S1**). Meanwhile, three fruits and four leaves were randomly collected from each plant. Single fruit fresh weight, fresh seed weight, leaf water content, and seed water content were measured according to the method of Zhang et al. (2021) and Lu et al. (2025). Day 0 of the treatment served as the control (CK). In addition, current-year leaves and seeds with the outer seed coat removed were collected in triplicate at each time point, immediately frozen in liquid nitrogen, and stored at −80 °C for gene expression analysis and physiological indicators. The physiological indicators tested in this study include malondialdehyde (MDA), H_2_O_2_, proline, and peroxidase (POD) (Wang, 2024).

### 2.6 RT-qPCR

Total RNA was isolated using the Plant RNA Kit (R6827, OMEGA, USA), and reverse transcription was performed with the HiExact RT SuperMix for qPCR (+gDNA Wiper) (RE523, Vazyme, Nanjing, China) to synthesize first-strand cDNA. The primers were designed using Primer Premier 6.0 software (**Supplementary Table S2**), and *CoEF1α* was used as the reference gene (Song et al., 2023). The reaction system was prepared according to the instruction of ChamQ Universal SYBR qPCR Master Mix (Q711, Vazyme, Nanjing, China). The reaction of RT-qPCR was carried out on a CFX96™ Real-time PCR system (BIO-RAD, USA). The relative gene expression levels were calculated by the 2^−ΔΔCT^ method (Livak and Schmittgen, 2001).

### 2.7 Statistical analysis

All experimental data are presented as the mean ± standard deviation (SD) of three biological replicates. The difference analysis in data was performed by Duncan’s test using SPSS 30.0 software (*p* < 0.05). All data were analyzed by one-way analysis of variance (ANOVA) using SPSS 30.0 software to test for differences among treatment groups. Following a significant ANOVA, Duncan’s multiple range test (*p* < 0.05) was used for pairwise comparisons among treatments. It should be noted that this test method does not require multiple comparison correction.

## 3 Results

### 3.1 Identification of the *C. oleifera* AQP family

A total of 166 *CoAQP* genes were identified from *C. oleifera*. A maximum likelihood tree was constructed with the amino acid sequences of CoAQPs, AtAQPs (Johanson et al., 2001), and PtAQPs (Lopez et al., 2012). Based on sequence homology, CoAQPs were grouped into five subfamilies, including CoPIPs, CoTIPs, CoNIPs, CoSIPs, and CoXIPs, with 65, 52, 31, 9, and 9 members, respectively. CoPIPs were further divided into two subgroups, including 30 CoPIP1 and 35 CoPIP2 members. CoTIPs were composed of five subgroups, namely 16 CoTIP1, 14 CoTIP2, two CoTIP3, three CoTIP4, and 17 CoTIP5 members. CoNIPs subfamily contained 18 CoNIP1, two CoNIP2, and 11 CoNIP3 members. Both CoSIPs and CoXIPs were each classified into two subgroups, consisting of five CoSIP1, four CoSIP2, four CoXIP1, and five CoXIP2 members, respectively. Based on sequence homology and chromosomal locations, all CoAQP members within each subfamily were named (**Figure 1**; **Supplementary Table S3**).

**Figure 1:**
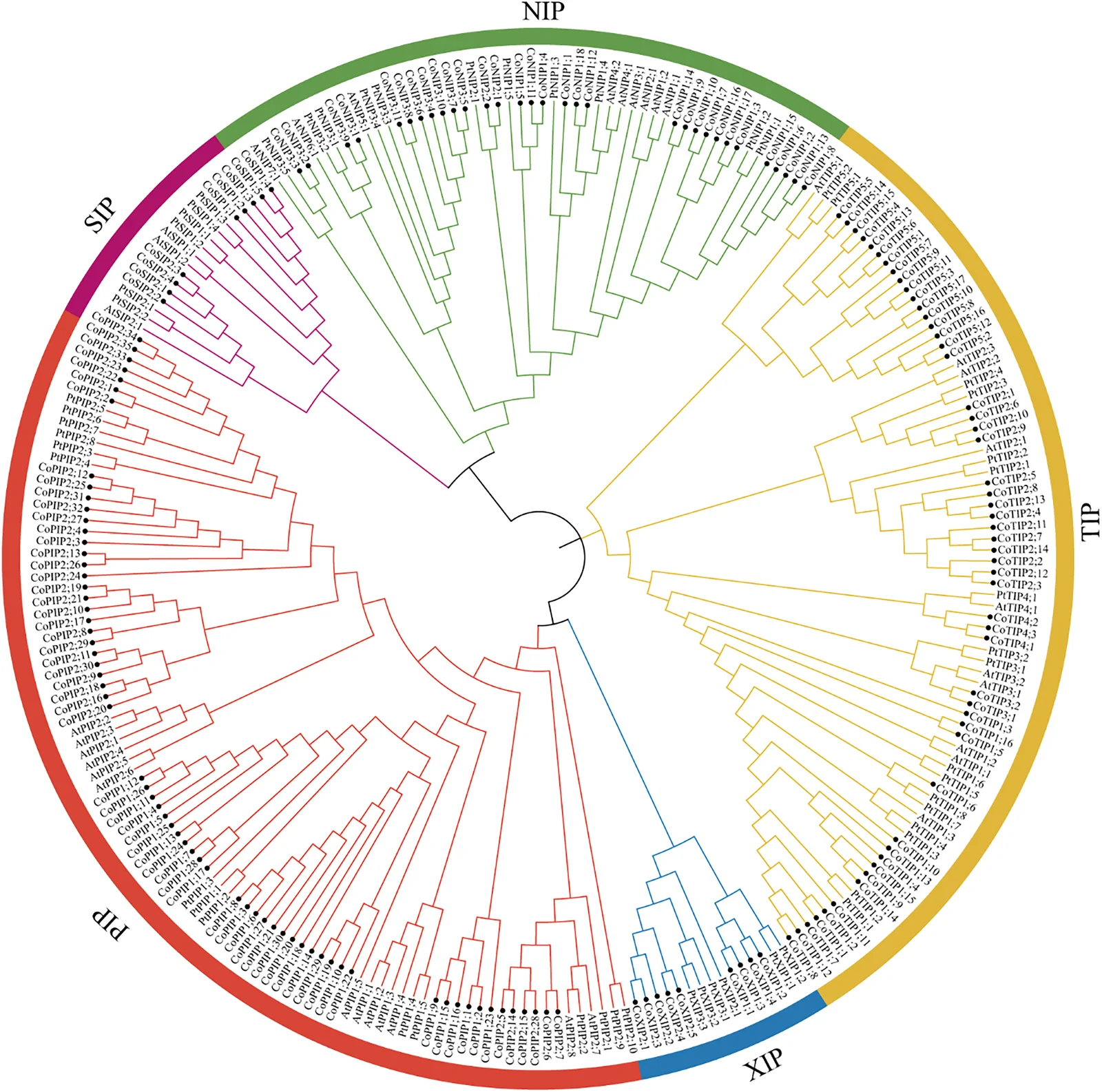
Phylogenetic analysis of CoAQPs from *C. oleifera*, AtAQPs from *A. thaliana*, and PtAQPs from *P. trichocarpa*. The phylogenetic tree was constructed using the maximum likelihood method with 1000 bootstraps. Five subfamilies of CoAQPs (CoPIPs, CoTIPs, CoNIPs, CoSIPs, and CoXIPs) clustered with the homologous AtAQPs and PtAQPs. The red, blue, brown, green, and purple clade represent the CoPIP, CoXIP, CoTIP, CoNIP, and CoSIP subfamilies, respectively.

The length and molecular weight of CoAQP proteins ranged from 57 aa (CoNIP3;4 and CoNIP3;6) to 1024 aa (CoPIP1;9 and CoPIP1;16) and 6.12 kDa (CoNIP3;4 and CoNIP3;6) to 111.28 kDa (CoPIP1;9), respectively. The theoretical pI ranged from 4.42 (CoPIP1;23) to 10.52 (CoNIP3;4 and CoNIP3;6). Among them, 105 CoAQPs were alkaline proteins with a theoretical pI higher than 7.0. The instability index of 139 CoAQPs was less than 40, suggesting they were probably stable proteins. A total of 158 CoAQPs might be hydrophobicity, because of their grand average of hydropathicity being higher than zero. All CoAQPs possessed TM domains, with 104 members containing six TM domains, and 31 members containing five TM domains (**Supplementary Table S3**).

### 3.2 Features and structures of CoAQP proteins

Potential phosphorylation sites for CoAQPs were predicted. A total of 150 CoAQPs contained all three types of phosphorylation sites, including serine (S), threonine (T), and tyrosine (Y), while the remaining 16 CoAQPs only contained S and T sites. The subcellular localization of CoAQPs was predicted using the WoLF_PSORT and Plant-mPLoc programs. Most CoAQPs were localized to the plasma membrane, while some members were distributed in vacuoles, nucleus, chloroplasts, cytoplasm, endoplasmic reticulum, and Golgi apparatus (**Supplementary Table S3**). The exon-intron structure of *CoAQPs* was analyzed, and most members within the same subfamily exhibited conserved structure characteristics. The intron number of *CoAQPs* ranged from zero to nine. Most *CoPIPs*, *CoTIPs*, and *CoNIPs* possessed three, two, and four introns, respectively. All *CoSIPs* possessed two introns. For CoXIPs, four members contained one intron, two members contain two introns, and three members was intronless (**Figures 2A, B**, **3A, B**).

**Figure 2:**
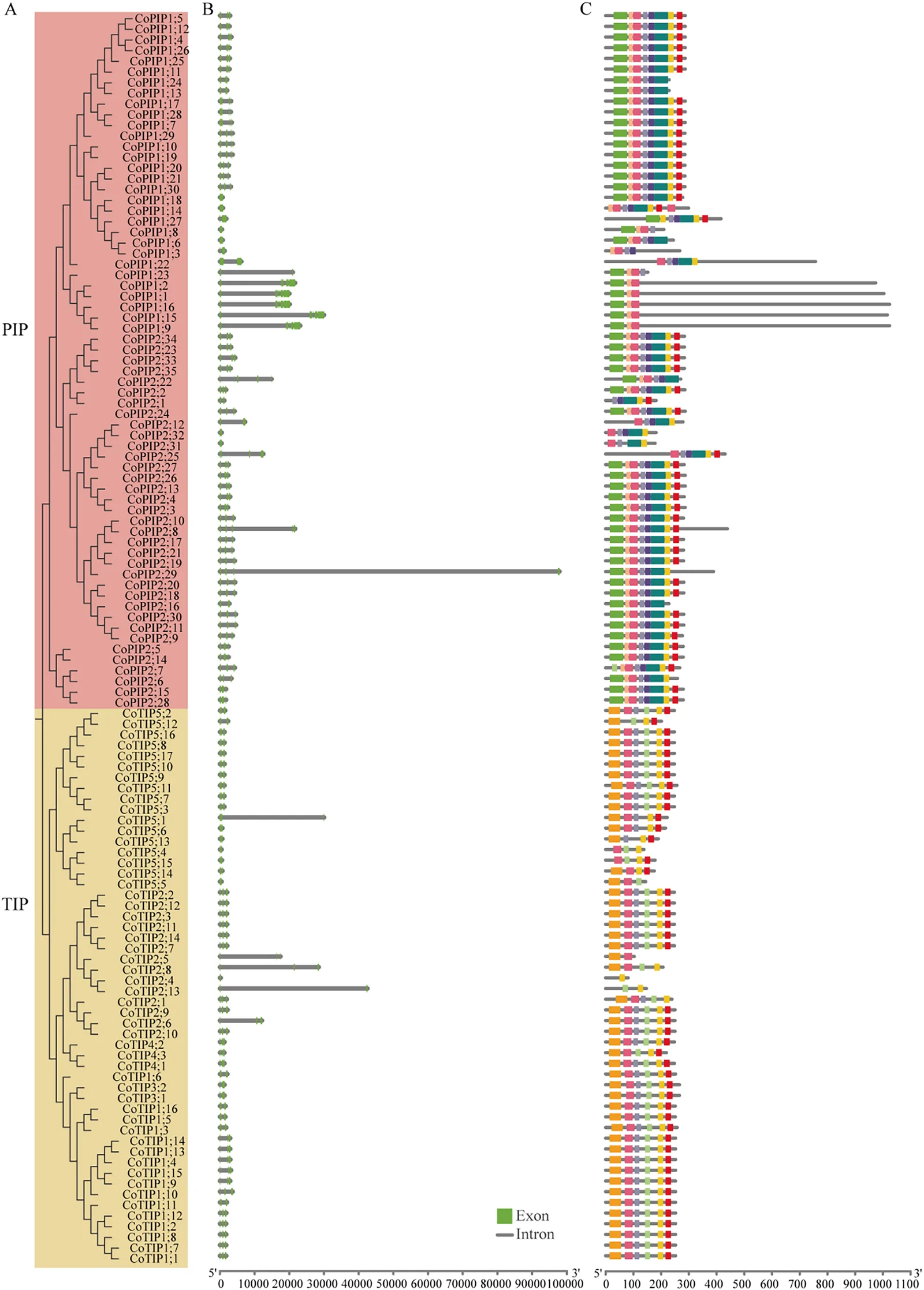
Phylogenetic relationships, gene structure, and motif compositions of CoPIP and CoTIP subfamilies in *C. oleifera*. **(A)** The phylogenetic tree was constructed by using MEGA11. CoPIP and CoTIP subfamilies are marked with different color backgrounds. **(B)** The exon-intron structure of CoPIP and CoTIP subfamilies. **(C)** The conserved motifs of CoPIP and CoTIP subfamilies identified by the MEME server. Different colored boxes represent different motifs. The motif logo is shown in **Supplementary Figure S1**.

**Figure 3:**
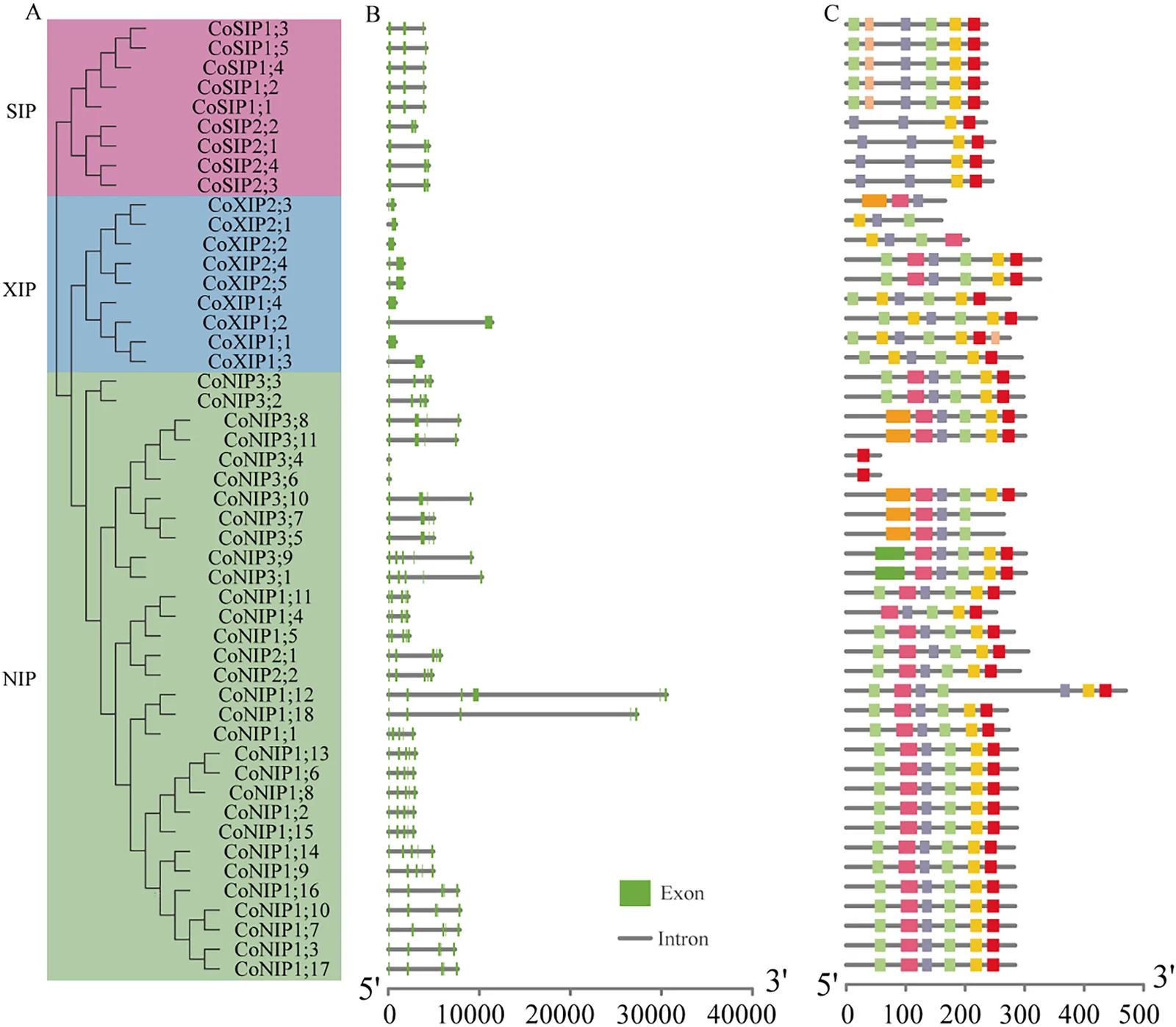
Phylogenetic relationships, gene structure, and motif compositions of CoSIP, CoXIP and CoNIP subfamilies in *C. oleifera*. **(A)** The phylogenetic tree was constructed by using MEGA11. CoSIP, CoXIP and CoNIP subfamilies are marked with different color backgrounds. **(B)** The exon-intron structure of CoSIP, CoXIP and CoNIP subfamilies. **(C)** The conserved motifs of CoSIP, CoXIP and CoNIP subfamilies identified by the MEME server. Different colored boxes represent different motifs. The motif logo is shown in **Supplementary Figure S1**.

Ten conserved motifs were identified in CoAQPs (**Supplementary Figure S1**), and members within the same subfamily exhibited conserved motif compositions. Motifs 6, 2, and 5 were present in most CoAQPs (129), indicating they were the basic motifs. However, some motifs showed subfamily specificity. For example, motifs 1, 4, and 9 were exclusively found in CoPIPs, whereas motif 8 was unique to CoTIPs. These motifs may serve as characteristic domains of CoPIPs and CoTIPs. Additionally, some motifs existed in multiple subfamilies. Motif 3 existed in CoPIPs, CoTIPs, and CoNIPs, and motif 10 was identified in CoPIPs and CoSIP1 (**Figures 2A, C**, **3A, C**).

### 3.3 Chromosomal locations and evolutionary characterization of *CoAQPs*

The chromosome localization map of *CoAQP* genes was constructed. The results showed that *CoAQPs* were distributed on 38 chromosomes, with 55, 67, and 44 members located in subgenomes A, B, and C, respectively. The distribution of *CoAQPs* on chromosomes was uneven, with the highest number of *CoAQPs* on chromosome B08 (17), followed by chromosome B12 (10). Notably, chromosomes A12, A14, A15, B01, B15, C01, and C15 only contained *CoTIPs*. *CoXIPs* were exclusively found in chromosomes A01, A02, A03, and B03, and *CoSIPs* were only present on chromosomes A10, B10, and C10 (**Figure 4**).

**Figure 4:**
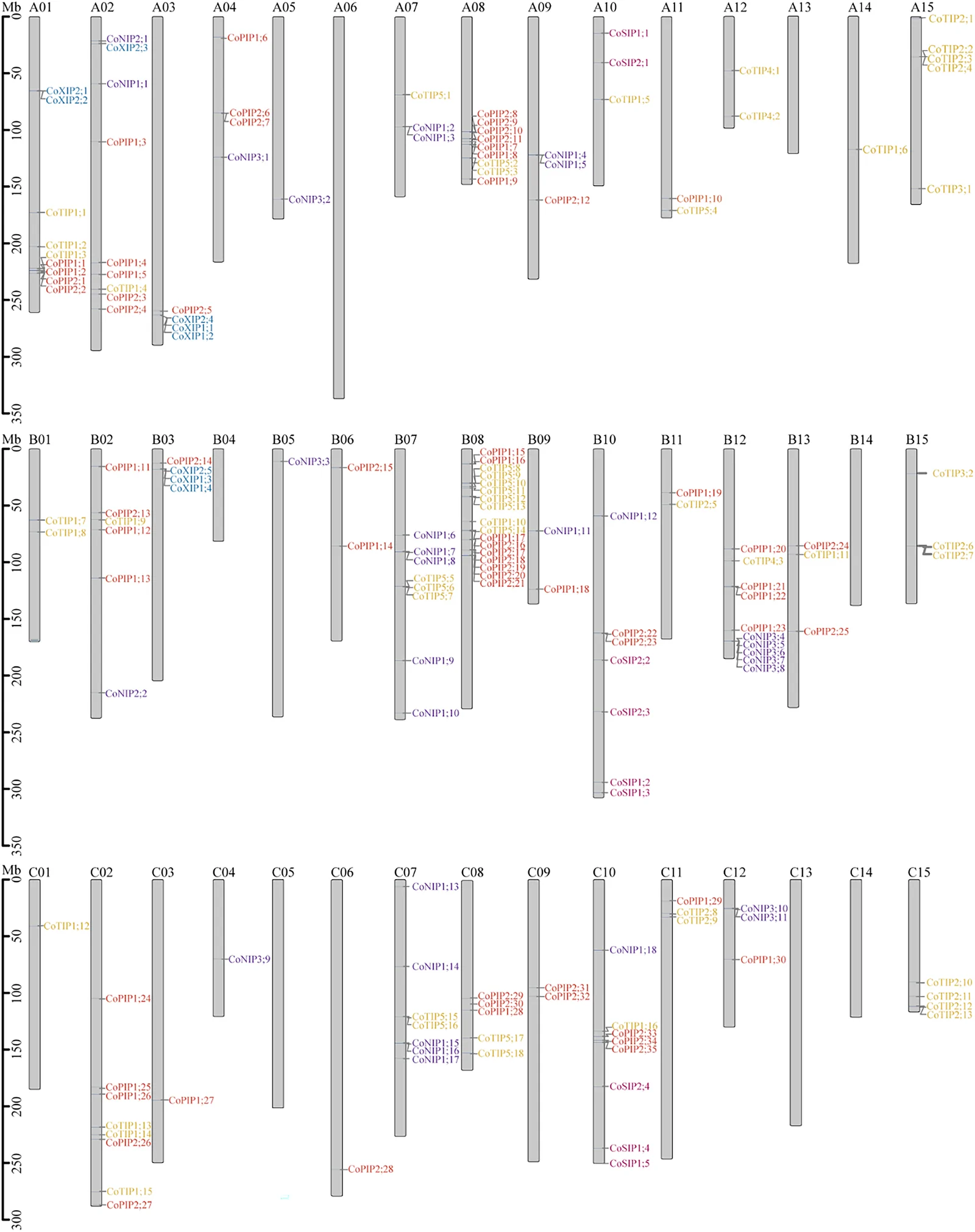
Chromosomal localization of *CoAQP* genes. Members of different subfamilies are indicated in different colors.

The gene duplication analysis was performed on *CoAQPs*. A total of 135 and nine *CoAQPs* were found to be segmentally and tandemly duplicated, respectively. Among these 135 *CoAQPs*, there were 215 segmental duplication events, with 91, 76, 30, 16, and 2 events occurring in 50 *CoPIPs*, 48 *CoTIPs*, 24 *CoNIPs*, 9 *CoSIPs*, and 4 *CoXIPs*, respectively. Four tandem duplication events were detected among these nine *CoAQPs* (**Figures 5A, B**; **Supplementary Table S4**). These findings indicated that segmental duplication acted as the major driving force for the evolution of *CoAQPs*. Ka/Ks ratios were further calculated for all duplicated gene pairs, with most Ka/Ks ratios being below one (**Figure 5C**; **Supplementary Table S4**), indicating that *CoAQPs* have undergone strong purifying selection.

**Figure 5:**
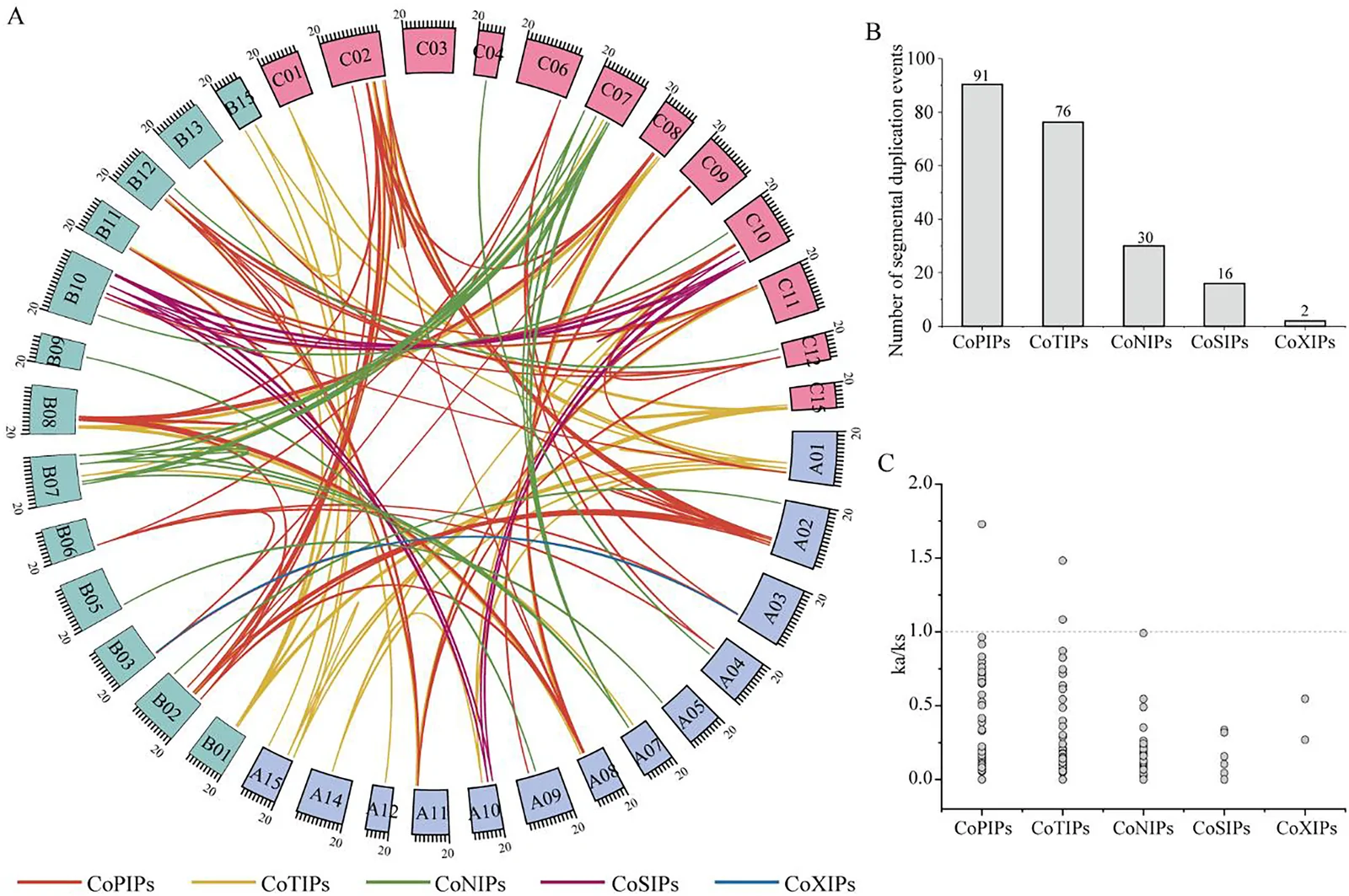
Segmental duplication of *CoAQPs* in *C. oleifera* chromosomes and Ka/Ks ratios. **(A)** The distribution of segmental duplication events of *CoAQPs*. **(B)** The number of segmental duplication events in *CoPIP*, *CoTIP*, *CoNIP*, *CoSIP*, and *CoXIP* subfamilies. **(C)** The Ka/Ks ratios of gene pairs of *CoPIP*, *CoTIP*, *CoNIP*, *CoSIP*, and *CoXIP* subfamilies.

### 3.4 Cis-acting elements analysis

A total of 69 types of cis-acting elements were identified in the promoters of *CoAQPs*, including 30 light responsive elements, four ABA responsive elements, two auxin responsive elements, two MeJA responsive elements, three gibberellin responsive elements, one ethylene responsive element, two SA responsive elements, ten abiotic or biotic stress-related responsive elements, and 15 development-related responsive elements. These elements were grouped into four categories, including the light responsive element, phytohormone responsive element, stress responsive element, and plant growth and development responsive element (**Figures 6**, **7**; **Supplementary Table S5**).

**Figure 6:**
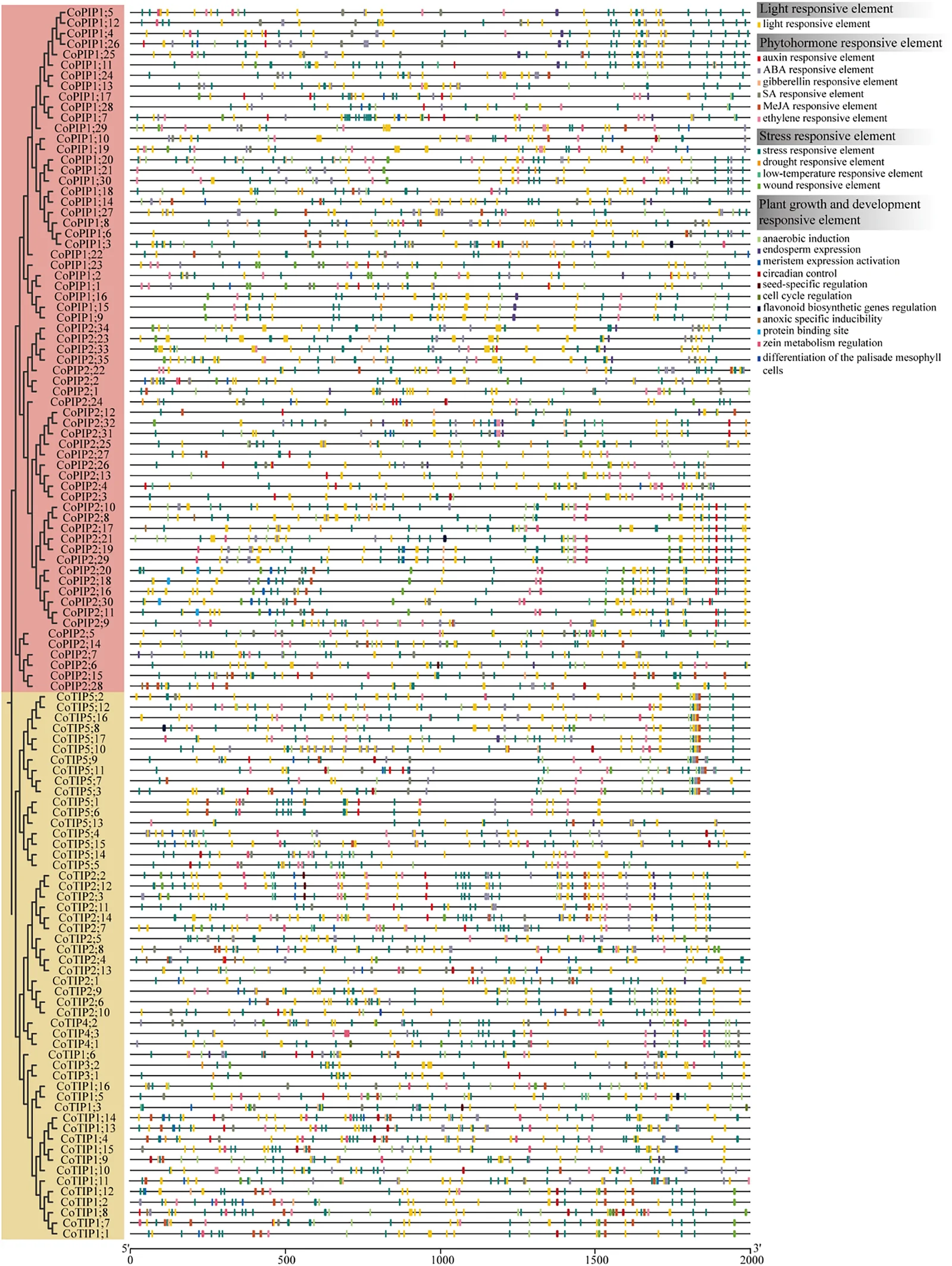
Distribution of the cis-acting elements in the promoter regions of *CoPIP* and *CoTIP* subfamilies. Each subfamily is marked with different color backgrounds. Different colored boxes represent different cis-acting elements.

**Figure 7:**
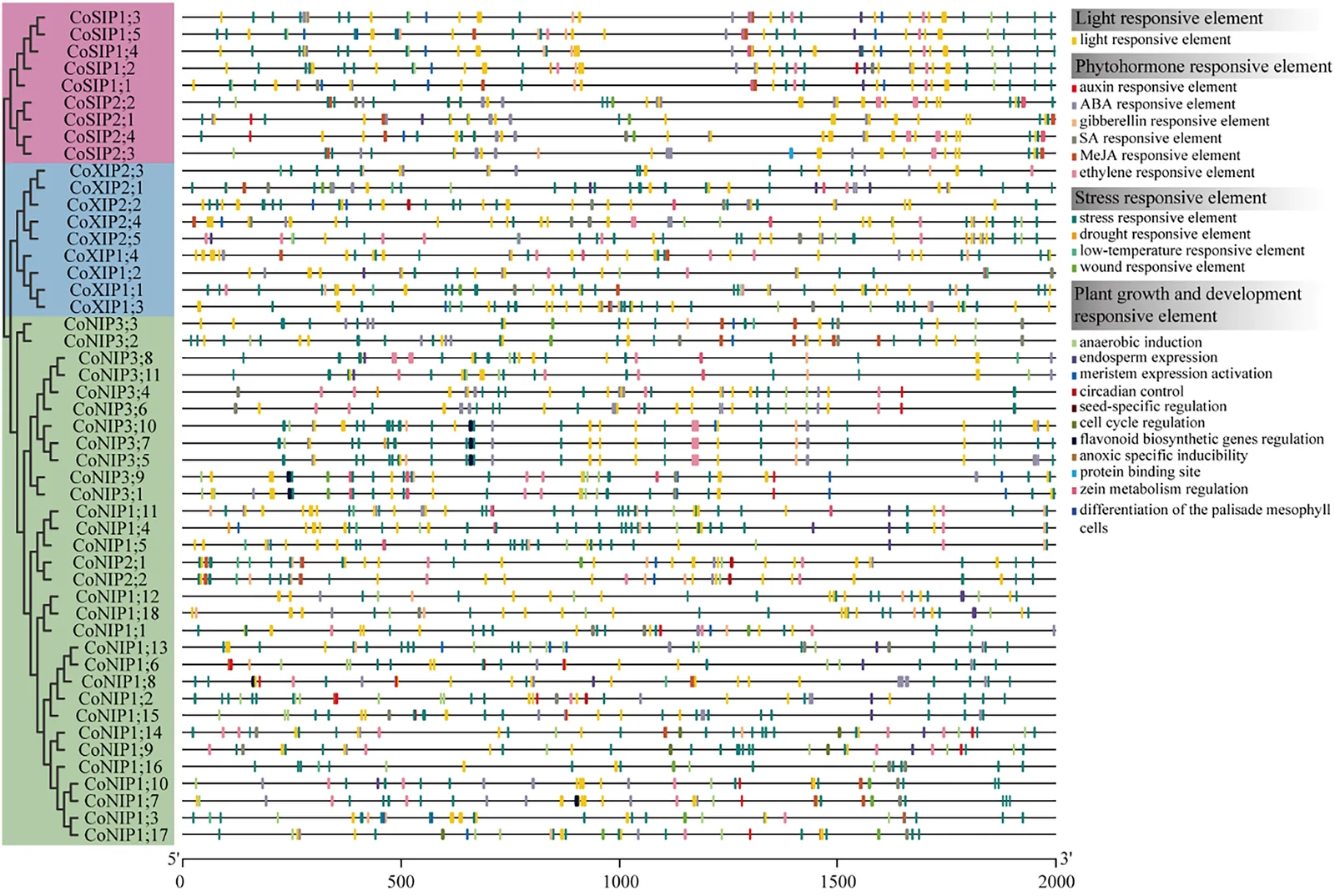
Distribution of the cis-acting elements in the promoter regions of *CoSIP*, *CoXIP*, and *CoNIP* subfamilies. Each subfamily is marked with different color backgrounds. Different colored boxes represent different cis-acting elements.

Light responsive elements were the most abundant, with a total of 1935 elements detected in all *CoAQPs* and an average of 11.66 elements per gene. Among phytohormone responsive elements, ABA responsive elements exhibited the largest quantity, with 652 elements detected in 149 genes and an average of 4.38 elements per gene. Among them, *CoTIP5;4* contained the highest number (21), followed by *CoTIP5;10* (16). There were 321 and 300 ethylene responsive elements and MeJA responsive elements, respectively, present in 129 and 95 *CoAQPs*. A total of 1918 stress responsive elements were identified, among which CoPIP1;11 (21) and CoTIP2;11 (20) possessed the highest element numbers. Furthermore, 141 wound responsive elements, 65 low-temperature responsive elements, and 63 drought responsive elements were also detected, which occurred in 87, 56 and 52 *CoAQPs*, respectively. For plant growth and development responsive elements, anaerobic induction elements were the most abundant (340), and were present in 129 *CoAQPs* (**Figure 6**, **7**; **Supplementary Table S5**).

### 3.5 Expression profiles of *CoAQPs* in different tissues and response to drought stress

The expression profiles of *CoAQPs* were detected in different tissues and under drought stress (**Figure 8**). Transcriptome analysis showed that 25 *CoAQPs* were highly expressed in pollen, among which *CoSIP2;4* exhibited the highest expression level, followed by *CoTIP5;10*. A total of 50 *CoAQPs* showed abundant expression in leaves, including 32 *CoPIPs*, eight *CoTIPs*, three *CoNIPs*, five *CoSIPs*, and two *CoXIPs*. Most *CoAQPs* were ubiquitously expressed in roots, with *CoPIP2;24*, *CoTIP1;3*, *CoPIP1;30*, *CoTIP1;5*, and *CoTIP1;11* displaying relatively high expression levels (**Figure 8A**). Oil synthesis in *C. oleifera* seeds initiate in late July, accompanied by a gradual decrease in seed water content (Zhang et al., 2021). Expression analysis of *CoAQPs* in seeds sampled from July to October demonstrated that 27 *CoAQPs* displayed a gradually decreasing expression trend, while *CoTIP3;1* was upregulated and was specifically expressed in seeds. Furthermore, they showed significantly higher expression levels in August and September (**Figure 8A**). Under drought stress, compared with the CK group, 12 *CoAQPs* were significantly downregulated in leaves at 2, 4, and 6 d, whereas *CoTIP1;3* was markedly upregulated at 4 and 6 d. In addition, with the prolongation of drought treatment, some members showed an upregulated or downregulated expression trend (**Figure 8B**). The above results indicate that *CoAQPs* may play crucial roles in the growth, development, and drought response of *C. oleifera*.

**Figure 8:**
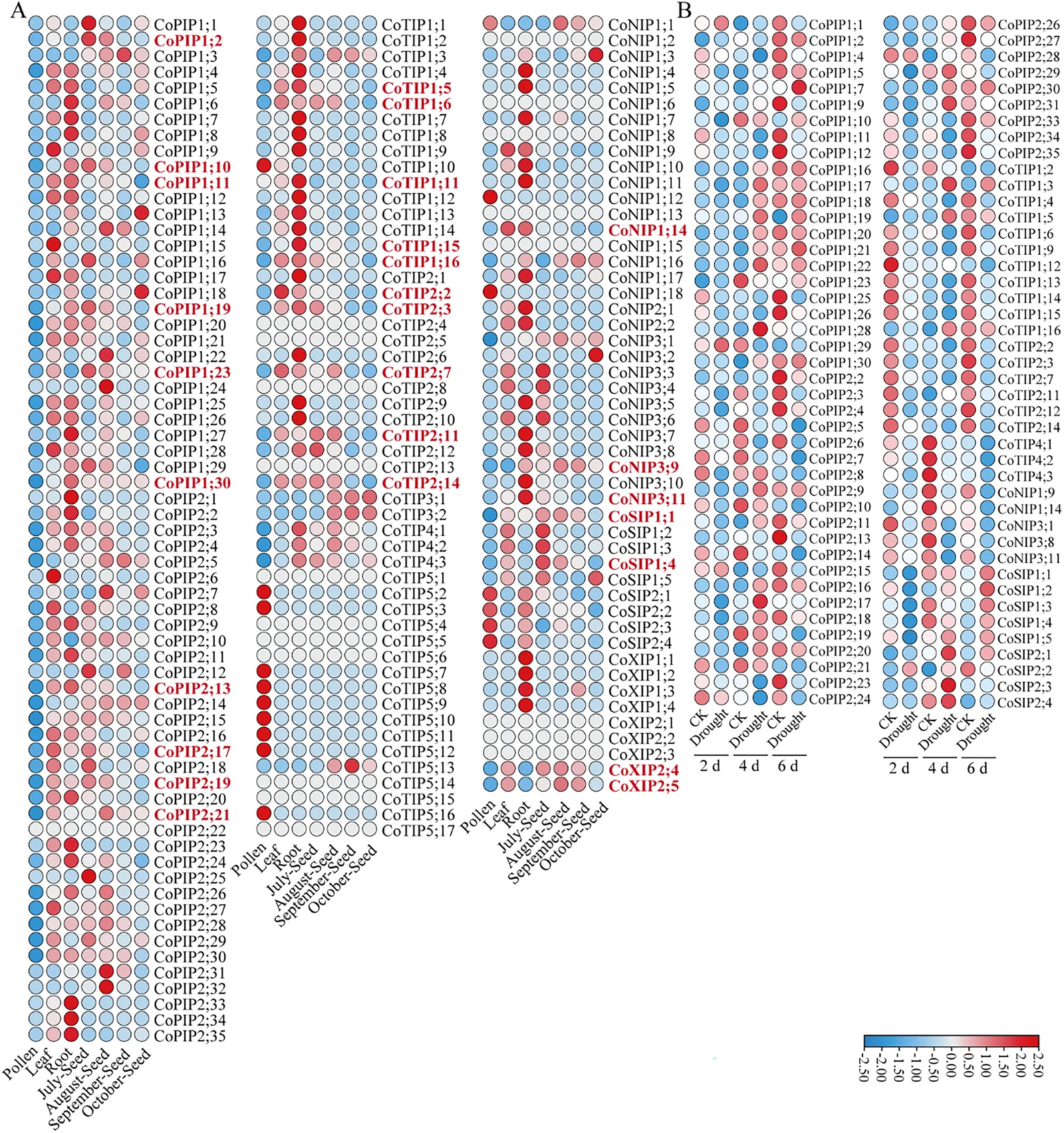
Expression levels of *CoAQPs* in different tissues **(A)** and leaves under drought stress **(B)** in *C. oleifera*. The data is normalized for each gene (row-normalized). Genes highlighted in red font represent members showing a gradually decreasing expression trend during seed development. The relative transcript accumulation is represented in a color code with blue and red showing respectively the lower and higher levels of transcript accumulation.

To verify the response of *CoAQPs* to drought stress, the drought treatment was carried out to *C. oleifera*. With the extension of treatment, the relative soil water content decreased significantly, reaching 16.97%-18.31% at 6 d of drought treatment (**Supplementary Table S1**). Meanwhile, leaves gradually turned brown and curled, accompanied by a continuous decrease in water content, which decreased by 24.75% from 0 d to 6 d. Physiological measurements of leaves revealed that the contents of MDA and proline increased progressively, rising by 82.60% and 324.19% at 6 d, respectively. H_2_O_2_ content increased significantly by 37.72% at 2 d. POD activity also increased significantly, peaking at 4 d, with an increase of 276.98% (**Supplementary Figure S2**). These results indicate that *C. oleifera* seedlings were subjected to drought stress, with the activation of antioxidant defense systems and osmotic adjustment mechanisms. Brown patches also formed on the fruit surface, and single fruit fresh weight, fresh seed weight, and seed water content all significantly decreased, with reductions of 71.10%, 65.74%, and 31.49% from 0 d to 6 d, respectively (**Figures 9A–C**). The expression levels of some *CoAQPs* in leaves and seeds were detected by RT-qPCR. In leaves, *CoPIP2;3* and *CoTIP1;3* was significantly upregulated on 4 d and 6 d of drought stress. By contrast, *CoPIP2;5*, *CoPIP2;14*, *CoTIP1;4*, *CoTIP1;6*, *CoTIP2;2* and *CoTIP4;1* showed downregulation on 2 d, 4 d, and 6 d (**Figure 9D**). In seeds, *CoTIP3;1* was significantly upregulated by drought treatment, and exhibited a trend of first increasing and then decreasing with prolonged drought treatment. *CoSIP1;5* and *CoXIP2;5* was significantly upregulated on 4 d and 6 d. Additionally, *CoPIP1;25*, *CoPIP2;13*, *CoTIP1;6*, *CoTIP2;2*, *CoTIP4;1* and *CoNIP1;1* was markedly downregulated on 4 d and 6 d (**Figure 9E**).

**Figure 9:**
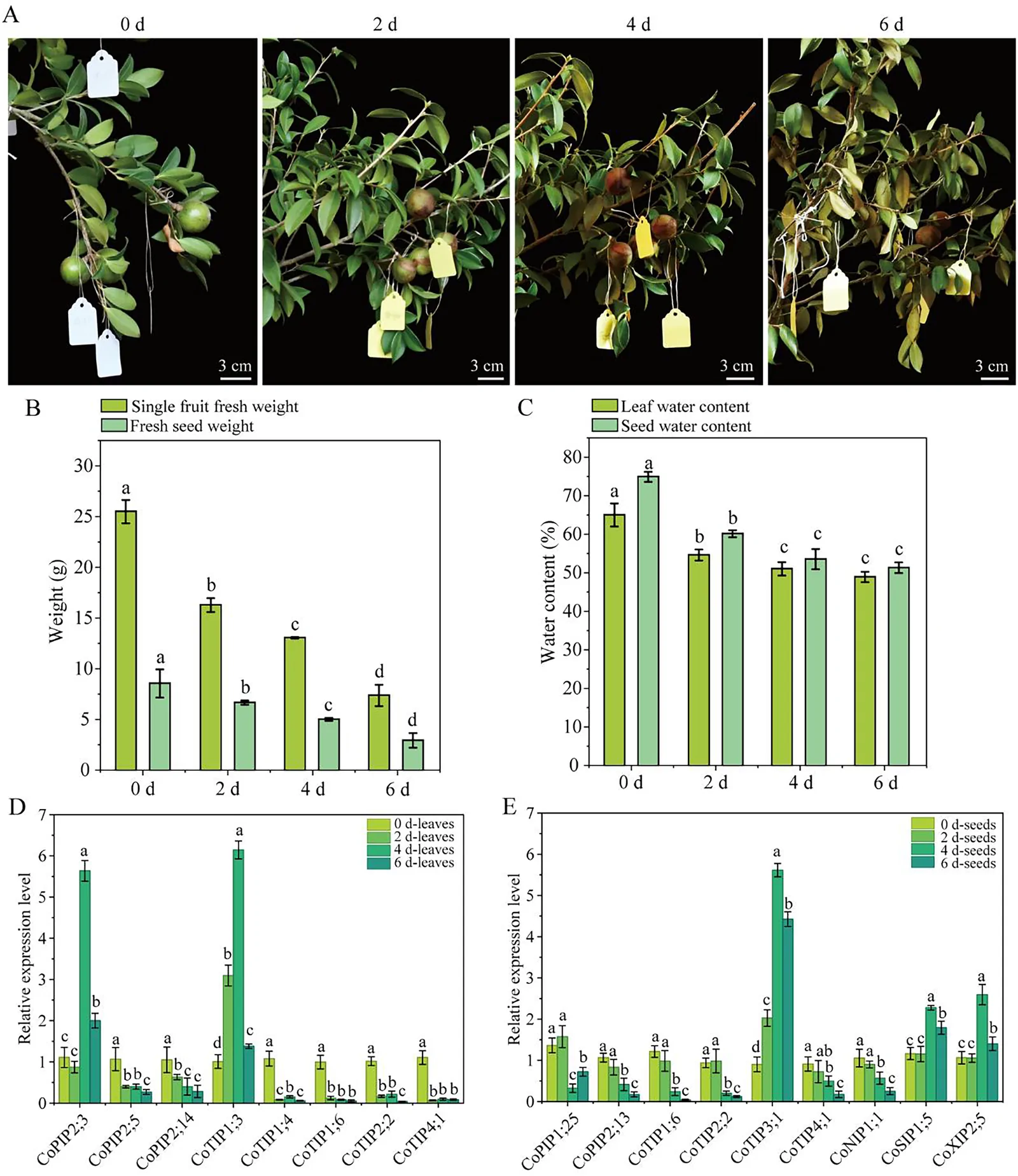
Growth status, water characteristics, and *CoAQPs* response of *Camellia oleifera* under drought stress. **(A)** Phenotypes of *C. oleifera* under drought stress. **(B)** Single fruit fresh weight and fresh seed weight of *C. oleifera* under drought stress. **(C)** Water content of *C. oleifera* leaves and seeds under drought stress. Expression levels of *CoAQPs* in *C. oleifera* leaves **(D)** and seeds **(E)** under drought stress. Different lowercase letters represent significant differences in the levels of the same indicator or gene expression at different times of drought stress (*p* < 0.05).

## 4 Discussion

Drought stress frequently occurs during the stage of fruit enlargement and oil synthesis accumulation, inhibiting plant growth and oil synthesis, and ultimately impairing the yield and quality of *C. oleifera*. Under drought stress, the expression of drought-related genes was induced to activate a series of physiological and biochemical responses, maintain intracellular water homeostasis, and improve drought adaptability of *C. oleifera* (Zhuang, 2008; Zhang and Liao, 2026; Liu et al., 2026; He et al., 2020; Dong et al., 2020; He et al., 2005). AQPs are transmembrane proteins that regulate water transport, and are necessary for plant drought response (Maurel et al., 2009). AQP gene family has been identified and studied in plants such as *O. sativa*, *A. thaliana*, *P. trichocarpa* (Maurel et al., 2015; Lopez et al., 2012). However, the research on AQPs family in *C. oleifera* remains limited. Herein, we performed genome-wide identification and systematic analysis of the *CoAQP* gene family, clarifying its gene structures, evolutionary patterns, and expression profiles. This study provides a theoretical basis and valuable genetic resources for comprehensively analyzing the drought response mechanism and exploring drought-tolerant and high-yield genes in *C. oleifera*.

A total of 166 *CoAQPs* were identified in *C. oleifera* (**Figure 1**), which was considerably more than the number of *AQPs* in typical diploid plants, such as *A. thaliana* (35), *O. sativa* (33), *Glycine max* (66), and *P. trichocarpa* (55) (Maurel et al., 2015; Lopez et al., 2012). The abundant expansion of the *CoAQP* family may be attributed to the hexaploid genome background of *C. oleifera* and the extensive segmental duplication events (**Figures 5A, B**). Meanwhile, 215 segmental duplication events were identified in the *CoAQP* family, with Ka/Ks values below one (**Figure 5**; **Supplementary Table S4**), indicating that the *CoAQP* family has undergone strong purifying selection during evolution, retaining highly conserved functions. In summary, these results suggest that segmental duplication serves as the major driving force for *CoAQP* family expansion, which is consistent with the findings of Lin et al. (2021).

Gene structure and its diversity are important indicators of gene function. Physicochemical analysis revealed that *CoAQPs* exhibited wide variations in gene length, molecular weight, theoretical pI, and number of TM domains. Most CoAQPs were hydrophobic (**Supplementary Table S3**), which was consistent with the characteristics of *KoAQPs* in *Kandelia obovata* (Guo et al., 2022) and *CrAQPs* in *Canavalia rosea* (Lin et al., 2021). Phylogenetic analysis based on the protein sequences of CoAQPs, AtAQPs, and PtAQPs classified the *CoAQPs* family into *CoPIPs*, *CoTIPs*, *CoNIPs*, *CoSIPs*和*CoXIPs* subfamilies (**Figure 1**). Within each subfamily, the exon–intron structure and motif distribution characteristic were highly conserved (**Figures 2**, **3**), indicating a common ancestral origin of the *CoAQP* subfamilies. This is consistent with the gene structure analysis of *KoAQPs* and *CrAQPs* reported by Guo et al. (2022) and Lin et al. (2021), respectively. Furthermore, protein phosphorylation is vital for the transport of water and small molecules, as well as for the subcellular localization of AQPs (Fox et al., 2017; Verdoucq et al., 2014). A large amount of phosphorylation sites was predicted in *CoAQPs*, and most members were speculated to localize to the plasma membrane and vacuole (**Supplementary Table S3**). Taken together, CoAQPs possess sequence features of the AQP family, however, the role of these features for AQP functional diversity requires further experimental investigation.

The cis-acting elements regulate the spatiotemporal expression patterns and stress response characteristics of genes. In this study, a total of 69 types of cis-acting elements were identified in the promoter regions of *CoAQPs*, including the light responsive element, phytohormone responsive element, stress responsive element, and plant growth and development responsive element (**Figures 6**, **7**; **Supplementary Table S5**). This result indicated the possible functional diversity of *CoAQPs* in regulating growth, development, and stress response in *C. oleifera*. The seed-specific regulation element was detected in the promoters of *CoTIP1;3*, *CoTIP2;2*, *CoTIP2;3*, and *CoTIP2;12*. In addition, the endosperm expression element was present in 15 *CoPIPs*, ten *CoTIPs*, four *CoNIPs*, six *CoSIPs*, and one *CoXIP* (**Figures 6**, **7**; **Supplementary Table S5**). Transcriptome data further confirmed that these genes were highly expressed in developing seeds (**Figure 8A**), implying their possible roles in seed development. Notably, stress responsive elements were universally distributed in the promoters of all *CoAQP*s (**Figures 6**, **7**; **Supplementary Table S5**), providing extensive candidate targets for further investigation of the CoAQP family in plant stress responses, although experimental validation is required to confirm their roles.

Gene expression profiles can also reflect the diversity of gene functions. Previous studies demonstrated that certain *AQPs* possess dual functions in regulating plant growth, development, and stress responses. For instance, apple *MdPIP1;3* exhibited a peak expression during fruit cell expansion and was significantly upregulated in leaves under drought stress. The overexpression of *MdPIP1;3* resulted in larger fruit size and stronger drought response in tomato plants (Wang et al., 2017). Soybean *GmPIP1;6* was highly expressed in pods, flowers and leaves, and its expression was induced by salt stress. Functional verification has confirmed that *GmPIP1;6* participates in root water transport, photosynthesis and seed development, thereby improving soybean salt tolerance and yield (Zhou et al., 2014). Here, drought stress caused leaf wilting and inhibited fruit development in *C. oleifera*, accompanied by significant decreases in leaf and seed water contents. Meanwhile, some *CoAQPs* were significantly upregulated or downregulated (**Figure 9**), indicating that the *CoAQP* family extensively participates in drought responses with distinct functional differentiation among individual members. The seeds of *C. oleifera* gradually mature from July to October, during which seed water content continuously decreases while oil content gradually increases (Zhang et al., 2021). Transcriptome analysis revealed dynamic expression changes of some *CoAQPs* during seed maturation. Notably, the seed-specific *CoTIP3;1*, which were homologous to *AtTIP3;1* and *AtTIP3;2* involved in seed maturation and germination in *A. thaliana* (Gattolin et al., 2011), exhibited the highest transcript abundance and were gradually upregulated during seed maturation (**Figures 1**, **8A**). These results suggest that *CoTIP3;1* may regulate seed maturation, oil biosynthesis and accumulation by maintaining seed water homeostasis, thereby serving as crucial regulators of yield and quality formation in *C. oleifera*. Additionally, the expression of *CoTIP3;1* was significantly upregulated in seeds under drought stress (**Figures 8B**, **9**). Overall, the expression profile of *CoTIP3;1* was consistent with a hypothesized dual role in seed maturation and drought adaptation. However, the hypothesized dual role of *CoTIP3;1* requires further confirmation through experiments such as protein expression analysis and transgenic functional verification.

## 5 Conclusions

In summary, a total of 166 *CoAQP*s were identified in *C. oleifera* in this study, and their physicochemical properties, structure characteristics, evolutionary mechanisms, cis-acting elements, tissue expression patterns, and drought responses were systematically analyzed. Gene structure and expression variations suggest broad involvement of *CoAQPs* in the tissue development and drought response in *C. oleifera*. Further expression analysis revealed that several *CoAQP* members exhibited hypothesized dual functions in regulating seed maturation and participating in drought response. These findings will enrich the evolutionary and functional research of AQP genes in woody oil plants, and provide a critical theoretical foundation for elucidating the regulatory mechanisms of water metabolism and exploring gene resources for stress tolerance and quality improvement in *C. oleifera*.

## Data Availability

The original contributions presented in the study are included in the article/**Supplementary Material**, further inquiries can be directed to the corresponding author/s.
