# Supplementary material for: Genome-wide identification of aquaporin family in Camellia oleifera and their response characteristics to drought stress
Source: Front Plant Sci. 2026 Sep 28;17:1959769. doi: 10.3389/fpls.2026.1959769 (PMC13654725; doi:10.3389/fpls.2026.1959769)
Supplement: Supplementary file 1 [file Supplementaryfile1.docx]

**Figure S1** The conserved motifs of *Camellia oleifera* AQP genes are identified by the MEME web server. The number of motifs was set to 10, and the E-value threshold was set to 1e-10. Different motifs are represented by different colored boxes.

**Figure S2** Changes in physiological indicators of *Camellia oleifera* leaves under natural drought stress. The contents of MDA (A), H_2_O_2_ (B), and proline (C) in leaves under drought stress. (D) POD activity in *C. oleifera* leaves under drought stress. Bars show mean values ± SD of n = 3 biological replicates.
